# Leaving the Hospital

**DOI:** 10.3201/eid1804.AD1804

**Published:** 2012-04

**Authors:** Anya Silver

**Keywords:** hospital, poem

As the doors glide shut behind me,

the world flares back into being—

I exist again, recover myself,

sunlight undimmed by dark panes,

the heat on my arms the earth’s breath.

The wind tongues me to my feet

like a doe licking clean her newborn fawn.

At my back, days measured by vital signs,

my mouth opened and arm extended,

the nighttime cries of a man withered

child-size by cancer, and the bells

of emptied IVs tolling through hallways.

Before me, life—mysterious, ordinary—

holding off pain with its muscular wings.

As I step to the curb, an orange moth

dives into the basket of roses

that lately stood on my sickroom table,

and the petals yield to its persistent

nudge, opening manifold and golden.

